# Fatal spontaneous rupture of common iliac artery associated with fibromuscular dysplasia

**DOI:** 10.1080/20961790.2016.1274467

**Published:** 2017-01-31

**Authors:** Xiang Xu, Mengchen Tsai, Ning Xiao, Xiaohui Tan, Fu Zhang, Yangeng Yu, Qi Wang, Weibing Xie, Huijun Wang, Dongri Li

**Affiliations:** a School of Forensic Medicine, Southern Medical University, Guangzhou, China; b School of Forensic Medicine, Wannan Medical College, Wuhu, China; c Department of Forensic Medicine, College of Medicine, National Taiwan University, Taipei, China; d Guangdong Public Security Department, Key Lab of Forensic Pathology, Guangzhou, China

**Keywords:** Forensic science, forensic pathology, fibromuscular dysplasia, abdominal aortic dissection, common iliac artery rupture, sudden death, forensic pathology

## Abstract

A previously healthy 25-year-old man with no known risk factors was presented at the emergency room with a 3 h history of abdominal and loin pain. Physical examination and lab data showed no specific findings except tenderness, slight white cell count elevation and decreased haemoglobin level. The patient's condition deteriorated over the following hours and he died despite resuscitation attempts. Autopsy revealed a 2.5-cm longitudinal tear in the intima of the right common iliac artery, which formed a thrombosed false lumen extending to the abdominal aorta proximally and to the left common iliac artery. Histopathologic examination revealed the characteristic changes of fibromuscular dysplasia (FMD). FMD involving the common iliac arteries is extremely rare; only six cases have been reported previously, and only two of those included forensic findings. The presented case is the first case of FMD with intimal tearing in the right common iliac artery, with propagation to the left common iliac artery and abdominal aorta. When a previously healthy young adult without hypertension or other risk factors presents with acute abdominal and loin pain, systemic vascular disease should be on the list of differential diagnoses. Careful and complete evaluation of multiple arteries can be critical.

## Introduction

Fibromuscular dysplasia (FMD) is a non-atherosclerotic, non-inflammatory vascular disease that mainly involves small- and medium-sized arteries, and that most commonly occurs in women between 30 and 50 years of age [1]. As the name implies, microscopic evaluation of affected artery walls reveals fibroplasia with disorganization of smooth muscle cells [2]. The aetiology of FMD is multifactorial and remains inconclusive. A number of aetiologies have been proposed, including environmental factors, such as smoking and oestrogen, as well as genetic factors. Previous studies have reported that approximately 10% of patients with FMD have an affected family member [3–5].

The diagnosis of FMD can be made with histopathology or angiography [6]. FMD is divided into three classifications based on the most affected layer of the arterial wall (intima, media or adventitia); each type has a distinctive pattern on imaging studies. Among the three types, medial FMD is the most common form, with the typical “string of beads” arteriographic appearance [7,8]. Affected arteries tend to be primarily occlusive; secondary events include aneurysm, dissection and arteriovenous fistula [9–11].

Although nearly every vascular bed in the body can be affected in patients with FMD, the renal arteries are most frequently involved (60%–75% of cases), followed by the internal carotid arteries (25%–30% of cases) [8,12,13]. FMD involving the common iliac arteries is extremely rare; to our knowledge, there have been six reported cases [9,14–18] (Table 1), only two of which have included forensic findings. We herein present a case of FMD with dissection of the right common iliac artery propagating to the left common iliac artery and abdominal aorta, which eventually led to fatal aortic rupture.

**Table 1.** Reports of iliac artery dissection dissection associated with fibromuscular dysplasia.

Author	Age (year)	Sex	Presentation	Site of dissection	Outcome
Burri et al. [19]	45	F	Left inguinal pain	Left external iliac artery	Alive
Sauer et al. [20]	56	F	Hip and thigh claudication	Right external iliac artery	Alive
Patel et al. [21]	39	M	Left inguinal pain	Left external iliac artery	Alive
Thevenet et al. [22]	45	F	Lower limb ischemia	Right external iliac artery	Alive
	51	M	Intermittent claudication	Bilateral external iliac arteries	Alive
	47	F	Abdominal pain	Right external iliac artery	Alive
	29	F	Iliac fossa pain	Left external iliac artery	Alive
	53	M	Intermittent claudication	Right external iliac artery	Alive
Lück et al. [23]	45	M	Intermittent claudication	Right external iliac artery	Alive
Honjo et al.[15]	30	M	Shock	Left common iliac artery	Dead
Yoshioka et al. [9]	21	M	Abdominal pain	Left common iliac artery	Dead
Akashi et al. [14]	49	M	Lower abdominal pain Intermittent claudication	Right common iliac artery	Alive
Teh et al. [16]	60	M	Right lower limb short-distance claudication Right buttock pain	Right common iliac artery	Alive
Savolainen et al. [17]	42	M	Groin pain and claudication	Right common iliac artery	Alive
Sugiura et al. [24]	30	M	Abdominal pain	Right external iliac artery	Alive
Hey and Röckelein [18]	38	F	Unknown	Splenic artery	Dead
Presented case	25	M	Abdominal and loin pain	Bilateral common iliac artery	Dead

## Case report

### Case history

A previously healthy 25-year-old man with no known risk factors (no smoking history) visited the emergency room with abdominal and loin pain of 3 h’ duration. The patient's vital signs were stable and physical examination showed no abnormality except tenderness in the right lumbar region of the abdomen and in the paravertebral muscles at L_4_–L_5_. Apart from a leukocyte count of 23.79 × 10^9^/L, haemoglobin of 10.8 g/dL and neutrophil ratio of 90.2%, haematological and laboratory values were within normal limits. Normal saline IV infusion and dezocine were administered, and the patient was kept under observation.

Four hours later, a change in consciousness and cyanosis of the lips and extremities were observed; heart rate and blood pressure could not be obtained. Bedside ultrasound showed accumulation of fluid with a hyperechoic mass in the abdominal cavity. Cardiopulmonary resuscitation (CPR), cardiac stimulants and vasopressors were administered, but the heart rate and blood pressure remained undetectable. The patient died despite resuscitation attempts. Because of the unclear circumstances of death, a medical‐legal autopsy was performed.

### Autopsy findings

**Figure 1.**
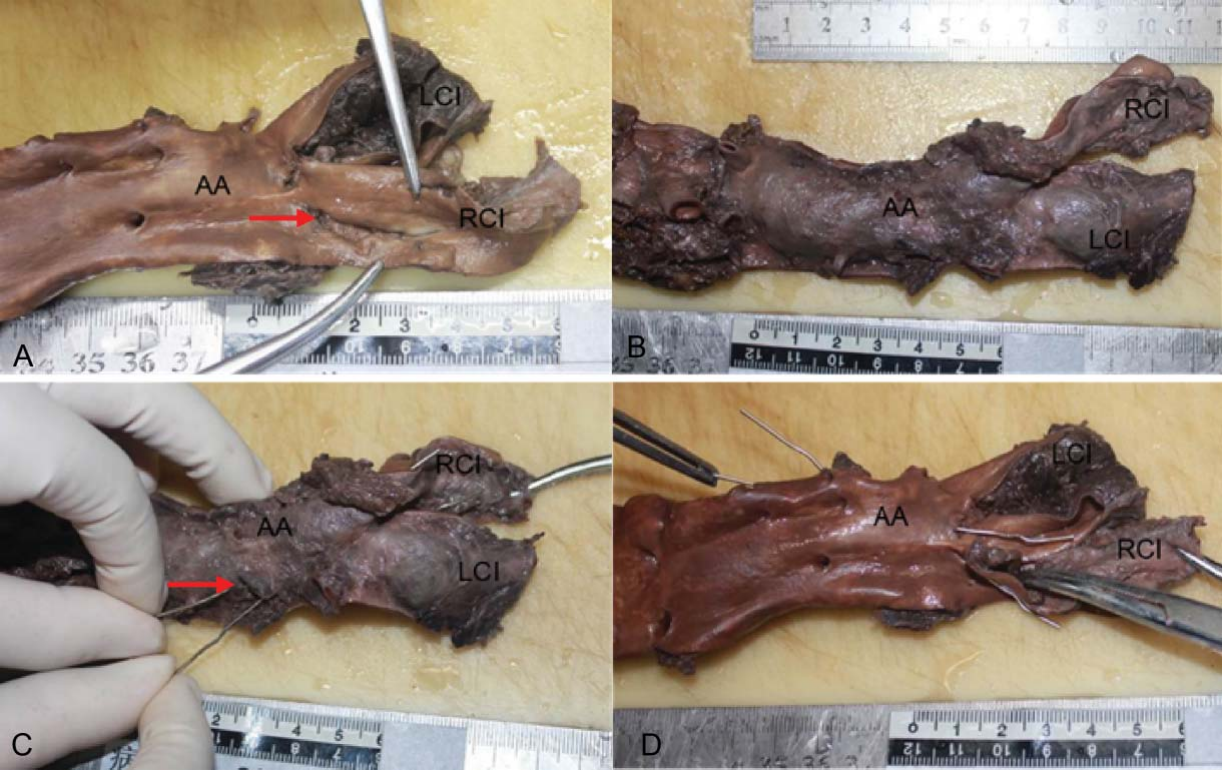
(A) A 2.5-cm longitudinal intimal tear (red arrow) was found in the right common iliac artery (RCI). (B) The thrombosed false lumen involved the abdominal aorta (AA) proximally and the left common iliac artery (LCI), forming a bulging appearance. (C) The 0.7-cm adventitial rupture of the abdominal aortic (AA) dissection (red arrow), 2.5 cm above the aortic bifurcation. The probes indicate communication between the adventitial rupture of the abdominal aorta (AA), dissection of the left common iliac artery (LCI) and the intimal tear of the right common iliac artery (RCI). (D) The luminal view, with the probes showing the communication.

Autopsy was performed on the day after death. No obvious abnormalities were observed on external examination apart from mild postmortem lividity. No unusual findings were noted in the head and neck region. With the exception of sternal fracture with surrounding soft tissue haemorrhage caused by CPR, there were no specific findings in the thoracic cavity. Five hundred millilitres of fluid blood and 1 100 g of clotted blood were present within the abdominal cavity. A 2.5-cm longitudinal tear was found in the intimal layer of the right common iliac artery, forming a thrombosed false lumen that involved the abdominal aorta proximally and the left common iliac artery (Figure 1(A)). The dissection extended 7 cm along the longitudinal axis of the abdominal aorta and 4.5 cm along the left common iliac artery (Figure 1(B)). The source of bleeding was a 0.7-cm adventitial rupture of the abdominal aortic dissection, located 2.5 cm above the aortic bifurcation (Figure 1(C,D)).

Grossly, no atheromatous plaques were found in the aorta or iliac arteries. The heart showed slight hypertrophic change (390 g) with no valvular degeneration or coronary artery anomaly. Other organs had no pathological changes or injuries aside from pallor.

### Histopathology

All tissue samples were fixed in formalin and embedded in paraffin. Aorta, vertebral arteries, celiac trunk, superior mesenteric artery, renal arteries and common iliac arteries were stained with haematoxylin–eosin (HE) stain, Masson's trichrome stain and elastica van Gieson (EvG) stain. Other organ sections were stained with HE.

**Figure 2.**
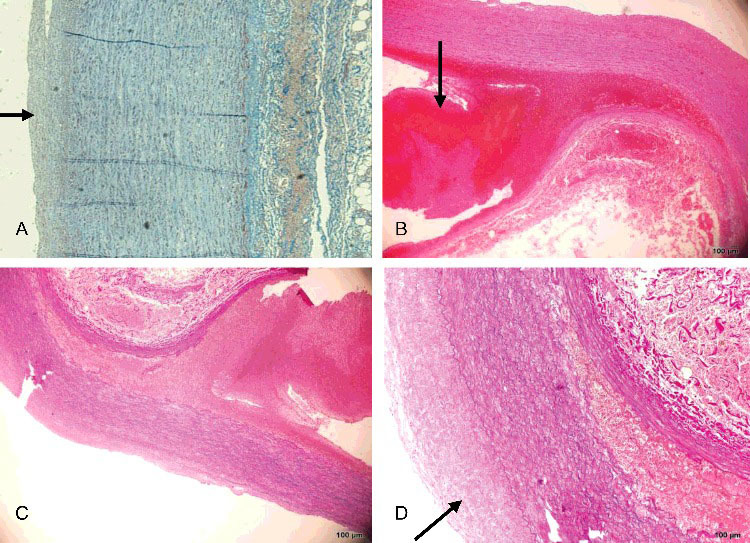
(A) The intact part of the aortic wall showed no histopathological change except slight intimal thickening (Masson trichrome×5). (B) Dissecting aneurysm of the abdominal aorta with longitudinal splitting of the vascular wall, ruptured adventitial layer and mural thrombus (black arrow) (HE×4). (C) Dissecting aneurysm of the abdominal aorta with longitudinal splitting of the vascular wall and splitting of elastic fibres (EvG×4). (D) Higher-power view of dissecting aneurysm of the abdominal aorta with evident thickened intima (black arrow) (EvG×10).

**Figure 3.**
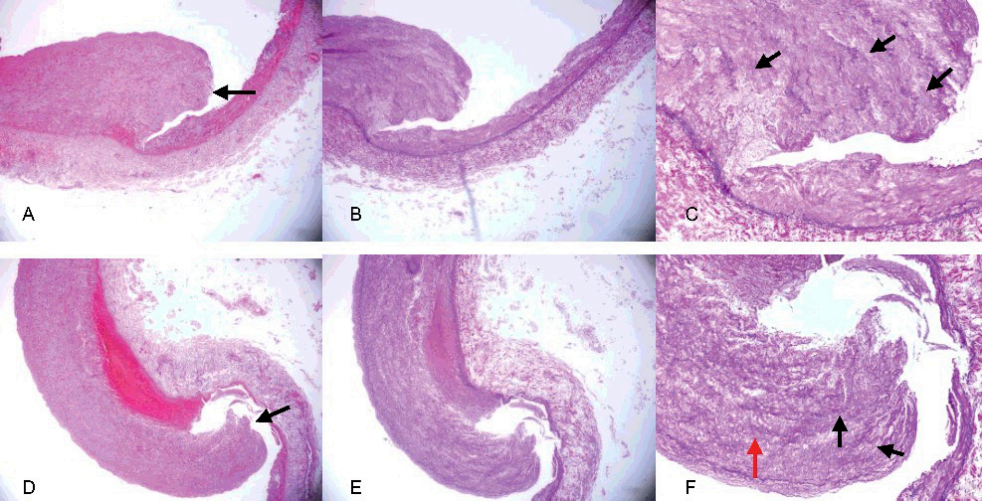
(A, D) Area of intimal layer rupture in the right common iliac artery (black arrow) showing hyperplasia of the intimal layer and depletion of smooth muscle cells, which are replaced with maloriented fibrous tissue (HE×4). (B, E) Area of intimal layer rupture in the right common iliac artery showing hyperplasia of the intimal layer, discontinuity of the internal elastic lamina, only scant rarefaction and fragmentation of elastic fibres and depletion of smooth muscle cells, which are replaced with maloriented elastic fibres and fibrous tissue (EvG×4). (C, F) Higher-power view of the right common iliac artery seen in (B, E) better illustrating the fibromuscular proliferation (red arrow), with rarefaction and fragmentation of elastic fibres (black arrow) in the muscularis and thinned areas of the vessel wall (EvG×10).

With the exception of slight intimal thickening, the intact part of the aortic wall showed no histopathological change (Figure 2(A)). In contrast, a ruptured adventitial and mural thrombus was observed at the aortic dissection site (Figure 2(B,C)), with evident thickening of the intimal layer and attenuated elastic fibres (Figure 2(D)). Compared with the aortic dissection site, the intimal rupture site in the right common iliac artery showed hyperplasia of the intimal layer and depletion of smooth muscle cells, which were replaced with maloriented elastic fibres and fibrous connective tissue (Figure 3(A–F)). There was lack of continuity of the internal elastic lamina. The smooth muscle nuclei at the rupture site showed mild enlargement and hyperchromatism, and had a block-like shape. Smooth muscle cells lay within a characteristic loose myxoid stroma of increased ground substance.

**Figure 4.**
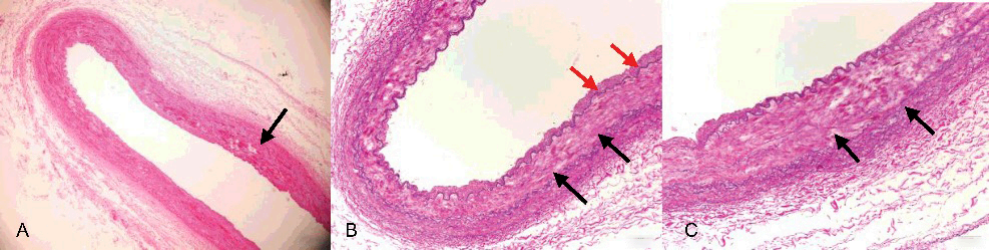
(A) Renal artery vessel alterations were seen in the media in areas with thick intima and fibroplasia with disorganization of smooth muscle cells (black arrow) (HE×4). (B) In the renal arteries, alterations in the media were seen in areas with thick intima (red arrows), fibroplasia with disorganization of smooth muscle cells and loss of elastica in the muscularis (black arrows) (EvG×4). (C) Higher-power view of the renal artery showing fibroplasia with disorganization of smooth muscle cells and loss of elastica in the muscularis (black arrow) (EvG×10).

**Figure 5.**
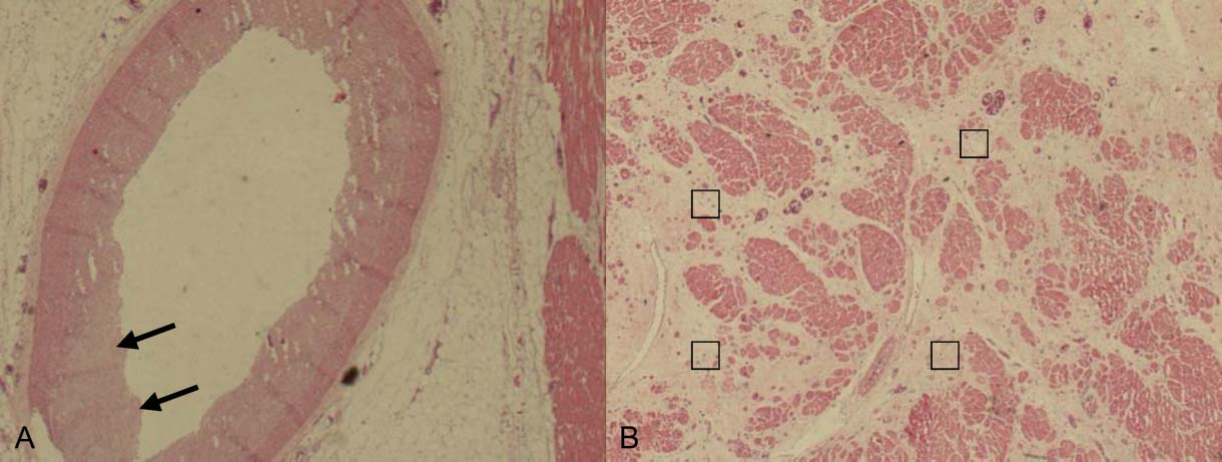
(A) The left anterior descending artery showed slight intimal thickening and internal elastic lamina discontinuity (black arrows) (HE×2). (B) Interstitial fibrosis (□) with capillary proliferation of left ventricle (HE×2).

Considering the typical histopathological characteristics of FMD, sections of the vertebral arteries, celiac trunk, superior mesenteric artery and renal arteries were submitted for microscopy. With the exception of the fairly normal structures of the vertebral arteries, all arteries showed mild intimal thickening and internal elastic lamina discontinuity. Thickening of the intima, fibroplasia with disorganization of smooth muscle cells and loss of elastica in the muscularis layer were observed in the renal arteries (Figure 4(A–C)). The left anterior descending artery, left circumflex artery and right coronary artery showed similar changes, with intimal thickening and internal elastic lamina discontinuity (Figure 5(A)). Furthermore, a regional decrease in myocytes and interstitial fibrosis with capillary proliferation were observed microscopically in the left ventricle and the ventricular septum (Figure 5(B)); these findings may be related to previous ischemic events.

### Cause of death

The cause of death was severe blood loss resulting from spontaneous rupture of the abdominal aorta and bilateral common iliac artery dissection attributed to FMD.

## Discussion

Acute aortic dissection is a medical emergency with high morbidity and mortality. Hypertension and atherosclerosis appear to be common risk factors in typical cases, which are men in their 60s and 70s [25]. Rarely aortic dissection occurs in individuals who seem outwardly healthy, but who are affected by congenital connective tissue disorders, such as Marfan syndrome, Ehlers–Danlos syndrome, or as in our case, FMD [26–28].

The prevalence of FMD in the general population is less than 1%, and this reflects only the symptomatic form. The clinical manifestations of FMD are determined by the artery affected and the degree of impairment of arterial blood flow. Involvement may be isolated to a single artery or may be generalized, involving multiple vascular beds concomitantly. A previous study demonstrated involvement of two or more arteries in 26% of patients with FMD [29]. Because the renal arteries are most often affected, renovascular hypertension is usually the first (and only) finding on physical examination [3,30]. The common iliac arteries are a less common site for FMD; inguinal pain, intermittent claudication, limb ischemia or abdominal pain may be present when these arteries are affected [11,15]. Once these abnormal symptoms occur, patients with FMD can be diagnosed with dedicated computed tomography angiography [31].

The patient in the present case had dissection of the right and left common iliac arteries as well as the aorta, but had no history of arterial hypertension, smoking or other risk factors for atherosclerosis. The patient had not experienced high-energy trauma, nor was he an athlete. Based on pathological examination, the aetiology in this case was FMD. In addition to the iliac arteries and the aorta, the celiac trunk, superior mesenteric artery, renal arteries and coronary arteries were affected. Irregular intimal widening, elastic fibre and smooth muscle cell reduction and disorganization, and internal elastic lamina degeneration were found in these vessels with different levels of severity.

As in our case, no specific symptoms or past medical history can guide us to the diagnosis of FMD, either in forensic or clinical practice. Therefore, we summarize some useful conclusions for forensic pathologists. Spontaneous dissection of the common iliac arteries or the aorta with rapidly progressive clinical symptoms in a previously healthy young adult without hypertension or other risk factors should raise suspicion of systemic vascular disease. In view of suspected medical error, a combination of conventional autopsy and postmortem imaging examinations, especially postmortem computed tomography angiography, can be performed to make a forensic diagnosis in these cases and to help the differential diagnosis [32–34]. Finally, because FMD is a generalized vascular disease, careful and complete inspection of multiple arteries can be critical; anatomical operation should be performed very cautiously to avoid secondary damage to ruptured vessels in the blood clot attachment area.

## Compliance with ethical standards

For this type of study formal consent is not required.
